# Next-generation sequencing of whole saliva from patients with primary Sjögren’s syndrome and non-Sjögren’s sicca reveals comparable salivary microbiota

**DOI:** 10.1080/20002297.2019.1660566

**Published:** 2019-08-29

**Authors:** Maria Lynn Sembler-Møller, Daniel Belstrøm, Henning Locht, Christian Enevold, Anne Marie Lynge Pedersen

**Affiliations:** a Section for Oral Pathology and Oral Medicine, Department of Odontology, Faculty of Health and Medical Sciences, University of Copenhagen, Copenhagen, Denmark; b Section for Periodontology and Microbiology, Department of Odontology, Faculty of Health and Medical Sciences, University of Copenhagen, Copenhagen, Denmark; c Department of Rheumatology, Frederiksberg Hospital, University of Copenhagen, Copenhagen, Denmark; d Institute for Inflammation Research, Center for Rheumatology and Spine Diseases, Copenhagen University Hospital, Copenhagen, Denmark

**Keywords:** Primary Sjögren’s syndrome, saliva, 16S rRNA, microbiota, hyposalivation

## Abstract

**Objective**: To characterize and compare the salivary microbiota in patients with pSS and patients with non-Sjögren’s-related sicca, and to relate the findings to their oral health status and saliva flow rates.

**Methods**: Twenty-four patients fulfilled the 2016 classification criteria for pSS and 34 did not (non-pSS). A clinical examination included registration of decayed, missing and filled teeth/-surfaces and collection of whole saliva. The microbiota was characterized using next-generation sequencing of the V1–V3 region of the *16S rRNA* gene. Data were annotated against the eHOMD database.

**Results**: A total of 509 different bacterial taxa were identified. There were no statistically significant differences between the groups with regard to the abundance of predominant genera, bacterial diversity and relative abundance on the genus or species level. The two groups did not differ with regard to general health, including intake of xerogenic medication and polypharmacy, oral health status or unstimulated and stimulated whole saliva flow rates.

**Conclusion**: The salivary microbiota and oral health status, as well as salivary flow rate in patients with pSS resemble that of non-pSS patients. Our findings indicate that changes in the salivary microbiota do not appear to be determined by the disease entity pSS itself.

Primary Sjögren’s syndrome (pSS) is a rheumatic autoimmune disease of unknown aetiology, which is characterized by lymphocytic infiltration of exocrine glands. It is particularly the salivary and lacrimal glands that are affected leading to hyposalivation and keratoconjunctivitis sicca and severe symptoms of oral and ocular dryness [1].

Saliva is a biological fluid, which has been widely investigated as a diagnostic medium, and several different salivary biomarkers have been suggested for pSS [2]. Additionally, saliva plays an important role in the maintenance of a natural balance between the host and the oral microbiota, which becomes evident in conditions with salivary gland dysfunction [3,4]. At low saliva flow rates, the salivary bicarbonate concentration drops, causing a reduction in salivary pH, and impairment of the salivary buffer capacity and clearance [5]. This can lead to a shift in the oral microbiota promoting colonization and growth of aciduric and acidogenic microorganisms, which increases the susceptibility for developing dental caries and oral candidiasis [5–7]. It has previously been shown that patients with pSS have a higher number of decayed, missing and filled teeth (DMF-T), as well as oral fungal infections, compared to healthy control subjects [7–11]. On the other hand, the risk of developing periodontitis does not appear to be higher in pSS [4,12].

Previous culture-based studies have shown higher counts of *Lactobacillus* and *Streptococcus mutans* in plaque and saliva from patients with pSS, and in patients with medication- and radiotherapy-induced hyposalivation, compared to healthy control subjects [13–17]. However, the use of culture-based microbial technique and selective media for identifying specific oral microorganisms has limitations and only display a minor fraction of the total oral microbiota found in the oral cavity [18,19].

A more recent study using high throughput sequencing of the *16S rRNA* gene has shown that reduced salivary secretion is associated with a bacterial shift in oral washings, while low relative abundance of *Streptococcus* was reported as being potentially disease-specific for pSS, irrespective of saliva flow rate and in comparison with sicca control and healthy control subjects [20]. Zhou et al. found a significantly higher relative abundance of *Veillonella* species in patients with pSS than in healthy control subjects, but no differences with regard to *Streptococcus* and *Lactobacillus* species [11]. On the other hand, Siddiqui et al. found a higher proportion of *Firmicutes* and *Streptococcus* species and a lower proportion of *Synergistetes* and *Spirochetes* in unstimulated whole saliva from pSS patients with saliva flow rates within the normal range compared to healthy control subjects. The authors suggest that pSS itself is associated with a characteristic microbial shift independent of hyposalivation [18]. Furthermore, our group has recently found a comparable salivary microbiota in patients with hyposalivation and patients with normal saliva flow rates, when both groups had comparable DMF-T and no active caries lesions present. This indicates that oral health status is a stronger determinant of the oral microbiota than the whole saliva flow rate per se [21].

Although there are few indications of a shift in the composition of the oral microbiota in pSS, it remains unclear whether this occurs as a result of pSS itself or more likely to occur as a result of disease-associated reduced salivary secretion. Moreover, it is unclear whether a specific shift in the oral microbiota makes patients with pSS more susceptible to develop oral and dental disease than patients with reduced salivary secretion of other causes.

We hypothesized that patients with pSS have a distinct salivary microbiota related to their salivary flow rates and reflected in a poorer oral health status compared to non-Sjögren’s syndrome-related sicca controls. The aim of this study was, therefore, to characterize and compare the microbiota in stimulated whole saliva samples from patients with pSS and patients with non-Sjögren’s syndrome-related sicca by means of next-generation sequencing (NGS). Furthermore, the aim was to compare the oral health status and saliva flow rates in the two groups.

## Methods and materials

### Study population

A total of 66 patients were screened of whom 62 were eligible for enrollment in the study. The inclusion criteria comprised the age from 18 to 75 years and the presence of symptoms of oral and/or ocular dryness. Exclusion criteria included pregnant and nursing women, use of local or systemic antibiotics 3 months prior to the examination and presence of secondary Sjögren’s syndrome. Four samples failed during the NGS analysis. Therefore, 58 patients were included in the study.

The patients were consecutively referred from rheumatology or ophthalmology out-patient clinics, private practising rheumatologists or dentists when a diagnosis of pSS was suspected. The patients underwent a diagnostic workup in accordance with the American College of Rheumatology/European League Against Rheumatism (ACR-EULAR) classification criteria [22]. Twenty-four patients fulfilled the ACR-EULAR classification criteria for pSS, while 34 patients (non-pSS) did not.

The study was conducted in accordance with the Declaration of Helsinki for experiments involving humans and approved by the Ethical Committees for the Region of Copenhagen, Denmark (H-160321 September 5289, 2016). Prior to the examination, all patients received oral and written information and written informed consent was obtained. The Danish Data Protection Authority has approved the establishment of a research biobank.

### Clinical examination and interview

The oral clinical examinations and collection of samples were performed by one examiner (MLSM) with assistance from a senior investigator (AMLP) from October 2016 through December 2017 at the Clinic of Oral Medicine, Department of Odontology at the University of Copenhagen. To avoid influence from diurnal variations in saliva secretion all procedures were performed at the same time of the day (10:00–12:00 a.m.). A standardized interview with a questionnaire regarding general and oral health was performed, including the presence of concomitant systemic diseases, daily intake of prescribed medication, symptoms of oral and ocular dryness, extraglandular manifestations, tobacco use and alcohol consumption.

Clinical examination included registration of DMF-T and -surfaces (DMF-S). Dental plaque, gingival inflammation and periodontal pocket depth were registered on six index teeth [23]. The oral mucosa was inspected for structural changes and external palpation of salivary glands and regional lymph nodes was performed.

### Measurement of saliva flow rates and collection of whole saliva samples

Unstimulated whole saliva (UWS) and paraffin-chewing stimulated whole saliva (SWS) flow rates were measured for 15 and 5 min, respectively, using the draining method [24]. Hyposalivation was defined as UWS ≤ 0.1 ml/min and SWS ≤ 0.7 ml/min (only UWS measurements were used for diagnostic workup), while a UWS flow rate at 0.3–0.4 ml/min and an SWS flow rate at 1.5–2.0 ml/min were considered the normal range [4].

Immediately after measurement of the SWS flow rate, each saliva sample was divided into different aliquots and placed on dry ice followed by storage in a −80°C freezer until further analyses. Bacterial characterization was performed using DNA-based identification with Human Oral Microbe Identification using *Next Generation Sequencing* (HOMI*NGS*).

### Sample preparation for NGS

The DNA extraction was carried out with 400 µl of chewing-stimulated whole saliva from all 62 patients, which was mixed thoroughly with 50 µl of a 50 mg/ml lysozyme solution (Cat. #90,082, ThermoFisher, Roskilde, Denmark) and incubated for 2 h at 37°C. Two samples failed due to too small volume and a deficient concentration of DNA. The entire sample was subsequently purified using the Maxwell 16 Cell DNA Purification Kit (Cat.# AS1020, Promega, Wisconsin) as instructed by the manufacturer. Purified DNA was measured using the Qubit dsDNA high-sensitivity kit (Cat. #Q32854, ThermoFisher, Roskilde) and normalized to 20 ng/µl.

### NGS and taxonomic assignment

HOMI*NGS* analysis was performed at the Forsyth Institute, Cambridge, MA. Sixty samples went through quality control by measuring DNA concentration using NanoDrop. PCR-amplification was performed with 50 ng (5 ng/µl in 10 µl) of the sample DNA using custom universal 16S primers targeting the highly conserved V1–V3 region of the *16S rRNA* gene. Purification was made using AMPure XP beads (Beckman Coulter). Amplicons from 60 samples were pooled in equal amounts of libraries into one tube with 100 ng/library, followed by gel-purification using the MinElute Gel Extraction Kit (Qiagen). Then, quantification using NEBNext Library Quant Kit and sequencing with Illumina® MiSeq^TM^ were performed. Two samples failed sequencing due to less than 7,500 reads (cut-off).

### Data analyses

The demographic and clinical data in Table 1 were processed in SPSS software and compared using unpaired t-test if data showed a normal distribution and non-parametric Mann–Whitney *U* test if not. Categorical data were tested with Fisher’s exact test. A P-value <0.05 was considered statistically significant for all purposes.

**Table 1.** Demographic and clinical data of pSS and non-pSS patients.

	pSS (*n* = 24)	non-pSS (*n* = 34)	*p* Value < 0.05
Age (Years)§	56 ± 10	54 ± 15	N.S.
Gender (F/M)	22/2	30/4	N.S.
Current smoker (yes/no)	6/18	7/27	N.S.
No. of prescribed medications*	1 (0–4)	1 (0–11)	N.S.
Polypharmacy (≥5) (yes/no)	0/24	5/29	N.S.
No. of xerogenic medications*	0 (0–1)	0 (0–4)	N.S.
Xerostomia (yes/no)	17/7	34/0	0.001
Ocular dryness (yes/no)	17/7	25/9	N.S.
Hyposalivation (yes/no)	18/6	20/14	N.S.
Keratoconjunctivitis sicca (yes/no)	19/5	16/18	0.016
Anti-SSA positive (yes/no)	24/0	4/30	<0.0001
Focus score ≥ 1.0 (yes/no)	6/18	0/34	0.003
UWS (ml/min)*	0.04 (0–0.39)	0.07 (0–0.37)	N.S.
SWS (ml/min)*	0.46 (0.08–2.34)	0.78 (0.05–1.72)	N.S.
DMF-T*	17 (3–23)	15 (1–24)	N.S.
DT*	1 (0–7)	1 (0–5)	N.S.
MT*	1 (0–11)	1.0 (0–13)	N.S.
FT*	12 (1–21)	12 (1–23)	N.S.
DMF-S*	39 (3–112)	46 (1–118)	N.S.
Plaque index*	2.30 (0.20–11.20)	2.50 (0.50–6.00)	N.S.
Gingival index*	2.80 (0.70–5.80)	2.40 (0.50–8.00)	N.S.
Periodontal pocket depth*	2.20 (1.90–3.50)	2.30 (1.90–3.30)	N.S.

§Given as mean ± standard deviation. *Given as median (range). UWS; unstimulated whole saliva flow rate, SWS; stimulated whole saliva flow rate, DMF-T; decayed-missing-filled-teeth, DT; decayed teeth, MT; missing teeth, FT; filled teeth, DMF-S; decayed-missing-filled-surfaces (five surfaces per tooth). Hyposalivation: UWS ≤ 0.1 ml/min.

Raw files were processed using the DADA2 R package to identify and quantify the sequencing reads from the MiSeq software [25]. Taxonomy of the identified amplicon sequence variants was assigned using the RDP classifier algorithm [26] based on the eHOMD database [27]. The salivary bacterial composition was characterized and compared between the two groups by means of principal component and correspondence analysis and with regard to relative abundance and bacterial diversity. The analysis was carried out using the Mann–Whitney *U* test with Benjamini Hochberg’s correction for multiple dependent analyses. For these analyses, MeV version 4_9_0 and GraphPad Prism version 5 were used as statistical software.

## Results

### Demographic and clinical characteristics of patients

Table 1 summarizes the demographic data and the clinical characteristics of the patients. There were no statistically significant differences between the two groups with regard to age, smoking status and intake of xerogenic medication or polypharmacy (≥5 prescribed medications)[28]. Furthermore, there was no difference in oral health status in terms of UWS and SWS flow rates, DMF-T/-S, dental plaque, gingival inflammation or periodontal pocket depth. The non-pSS group had a median UWS flow rate below the cut-off value (0.07 ml/min) and a median SWS flow rate just above the cut-off value (0.78 ml/min). However, the non-pSS group displayed great inter-individual variations in saliva flow rates (Table 1). There was no difference with regard to the presence of hyposalivation, but xerostomia was more prevalent in the non-pSS group than the pSS group (P = 0.001).

### NGS data

**Figure 1.**
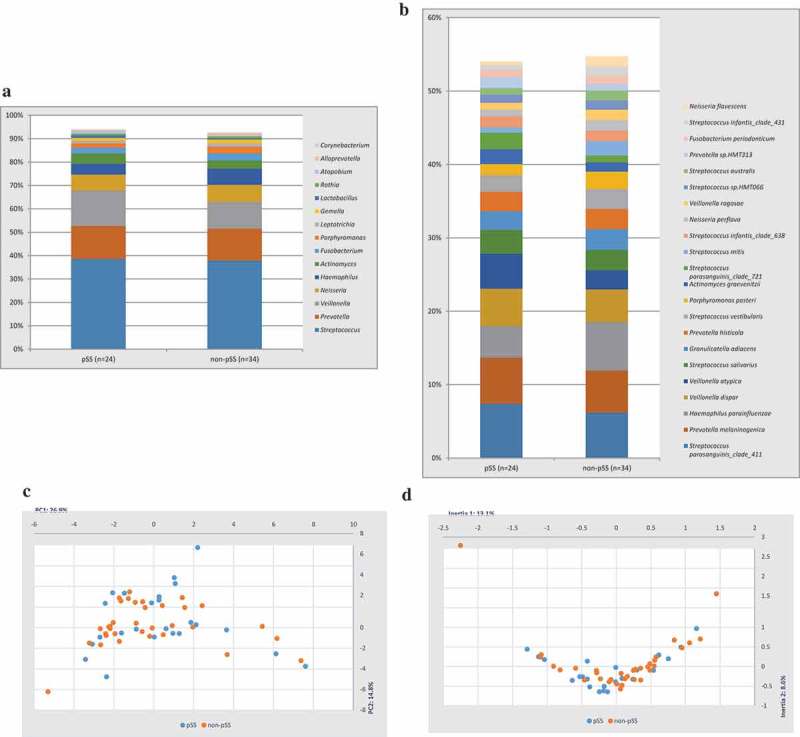
Comparative analysis on group level based on data from HOMI*NGS*. (a) Mean relative abundance of the 15 most predominant bacterial genera in each group. (b) Mean relative abundance of the bacterial species comprising >1% of the total microbiota from all 58 samples. (c) Principal component analysis, where axes are expressed as the two most decisive components accounting for a total of 41.7% of the variation in the dataset. (d) Correspondence analysis, where axes are expressed as the two most decisive inertias accounting for a cumulative inertia of 21.7%.

A total number of 1,404,081 sequences with a mean number of 24,208 (range 7,726–40,739) passed the quality control, from which a mean percentage of reads given at genus level was 99.9% (99.7–100%), while 98.6% (95.7–99.9%) could be identified at the species level. Less than 0.1% of the reads remained unassigned. In total, 509 different taxa were identified at the species level with a mean of 128 (range 33–213) bacterial taxa per sample. The predominant bacterial genera identified in both the pSS and non-pSS groups included *Streptococcus, Prevotella, Veillonella* and *Neisseria*, comprising 72% of the salivary microbiota (Figure 1(a)). There were no statistically significant differences between the pSS and non-pSS group in terms of the composition of bacterial genera and species (Figure 1(a–d)) and bacterial diversity (Shannon index 3.2 and 3.3, respectively, and P = 0.233).

## Discussion

In the present study, there were no differences between the two groups in terms of age, gender, general health, intake of xerogenic medication, oral health status, saliva flow rates and presence of hyposalivation. However, the non-pSS patients reported significantly more xerostomia than the patients with pSS. This may be ascribed to a higher prevalence of polypharmacy in this group, although not statistically significant. Our findings suggest that the cut-off value for hyposalivation at 0.1 ml/min can not be used to distinguish patients with pSS from non-pSS patients with Sjögren–like symptoms, as both groups had a median UWS below 0.1 ml/min (0.04 vs. 0.07 ml/min, respectively). Seventy-five per cent of the patients with pSS and 59% of the non-pSS patients had a UWS ≤ 0.1 ml/min, giving a low sensitivity at 47% and a specificity at 70%.

In this study, the patients with pSS and the non-pSS patients did not differ with regard to caries experience (DMF-T) or active caries lesions. In general, the number of active caries lesions was low, which may be ascribed to regular dental follow-up visits and regular daily use of fluoride-containing toothpaste in both groups. At the time of inclusion and examination, no specific dental preventive interventions had been initiated, as the patients were under diagnostic work-up for pSS, and thus the diagnosis was not finally confirmed.

A low incidence of active caries lesions in patients with pSS has been reported in previous studies as well [7,11,14], but also an association between pSS and a high incidence of dental caries [8,17,29–31]. A number of studies have shown a higher DMF-T score in patients with pSS than in healthy control subjects [7,9,11], indicating a high previous prevalence of caries. The DMF-T/-S indices provide solid and valid data on dental caries and are widely used, but also reflect a historical tradition of dental treatments. Thus, the implementation of prevention-oriented school health program several decades ago, the improved oral health behaviour, the regular use of fluoride, and the minimal invasive approach towards dental restorative treatment and indications for dental extractions are also seen in the DMF-T/-S scores of patients with pSS. Consequently, in this study, the mean number of DMF-S in the pSS group was 44 in contrast to 83 in a comparable study from 2005 [7].

The predominant bacterial genera identified in both the pSS and the non-pSS group included *Streptococcus, Prevotella, Veillonella* and *Neisseria*, comprising over 70% of the salivary microbiota. Thus, the composition of the salivary microbiota did not differ significantly between the two patient groups. These findings indicate that the salivary microbiota is not determined by the underlying disease of pSS per se, which are in line with the findings of a recent study by van der Meulen et al. [20]. In this study, the authors also suggest that reduced salivary flow is a determinant for a bacterial shift [20], but this is in contrast to another study indicating that the dental health status, rather than saliva flow rates, determines the salivary microbiota [21].

Other studies have shown that pSS is associated with a reduced bacterial diversity [11,18,32], but these findings are based on comparison to healthy control subjects.

In this study, we found no statistically significant difference in the relative abundance of *Streptococcus* species, which is in accordance with the findings of a recent study [11]. However, findings are conflicting, showing both lower [20] and higher proportions of *Streptococcus* species in patients with pSS [18,33], and none of these studies included measures on oral and dental health.

We have previously demonstrated that local disease can influence the salivary microbiota [34–36], which underlines the importance of recording oral and dental health status in relation to the interpretation of data on the oral and salivary microbiota.

Interestingly, none of the studies using the NGS technique found statistically significant differences of the cariogenic bacteria *S. mutans* and *Lactobacillus* species in saliva or oral washing in relation to pSS [11,18,20,32]. This questions the findings of previous studies using culture-based techniques, and indicates that selective multiplication of non-abundant species and genera might be misleading.

This study did not include a healthy control group, as the aim was to investigate whether patients with pSS have a distinct salivary microbiota distinguishing them from patients having Sjögren-like symptoms. Ideally, this can be useful in the diagnostic work-up and in the choice of the following therapeutic intervention.

In the future, it would be obvious to conduct a longitudinal study including a larger cohort of patients to investigate whether the salivary microbiota changes over time due to changes in bacterial activity, and if changes are related to oral and general disease state in pSS. Such a study would also enable assessment of the risk of developing oral disease in relation to pSS and hyposalivation. To assess the risk of developing caries, it would also be valuable to collect supragingival plaque samples from various sites in addition to whole saliva. A potential limitation of using stimulated whole saliva is that local oral bacteria are diluted when they become planktonic in saliva. Consequently, a bacterial shift of specific pathogenic bacteria might be discovered in a later stage in saliva than in a plaque sample. We have previously shown that local bacterial alterations in relation to oral hygiene discontinuation and non-surgical periodontal treatment are reflected in saliva [37,38], but the participants all had normal saliva flow rates. To the best of our knowledge, it remains to be demonstrated that the composition of the salivary microbiota reflects local bacterial alterations in patients with severe hyposalivation. Consequently, it may be questioned whether there are differences in the shedding of oral bacteria from oral sites related to the secretion of saliva from the various salivary glands. It may be speculated that bacteria are more adherent to oral surfaces in patients with pSS with markedly reduced salivary secretion as well as in patients with hyposalivation of other causes, and thus the saliva sample does not sufficiently reflect the bacterial composition and diversity in the oral cavity. Therefore, future studies of the salivary microbiota in patients with pSS should ideally also include local microbial samples.

In conclusion, the composition of the salivary microbiota did not differ between patients with pSS and non-pSS patients, nor did oral health status and saliva flow rates.

## Data Availability

Access to all NGS data will be granted upon request to mlse@sund.ku.dk.
